# Outcomes of surgical revascularization for pediatric moyamoya disease and syndrome

**DOI:** 10.1007/s00381-024-06393-w

**Published:** 2024-05-16

**Authors:** Jason H. Boulter, Nicholas S. Szuflita, Robert F. Keating, Suresh N. Magge

**Affiliations:** 1 Division of Neurosurgery, Walter Reed National Military Medical Center, Bethesda, MD USA; 2 Division of Neurosurgery, Children’s National Hospital, Washington, DC USA; 3 Department of Neurosurgery, University of Michigan, Ann Arbor, MI USA

**Keywords:** Moyamoya disease, Moyamoya syndrome, Indirect revascularization, Stroke risk

## Abstract

**Purpose:**

Moyamoya disease and syndrome represent rare entities characterized by progressive stenosis and/or occlusion of the intracranial blood vessels. We present our series of patients with moyamoya disease and syndrome stratified by underlying disease and analyze differences in presentation and outcome following surgical revascularization.

**Methods:**

This was an Institutional Review Board (IRB) approved, retrospective review of all patients surgically revascularized by the senior author (SNM) while at Children’s National Hospital in Washington, DC. Demographic data, presenting symptoms and severity, surgical details, and functional and radiographic outcomes were obtained and analyzed for differences among the underlying cohorts of moyamoya disease and syndrome as well as by unilateral or bilateral disease and index or non-index surgeries.

**Results:**

Twenty-two patients were identified with the following underlying diseases: six with idiopathic moyamoya disease, six with sickle cell anemia, five with trisomy 21, and five with neurofibromatosis type 1. Thirty hemispheres were revascularized with a significantly reduced rate of stroke from 3.19 strokes/year (SD = 3.10) to 0.13 strokes/year (SD = 0.25), *p* = 0.03. When analyzed by underlying cause of moyamoya syndrome, patients with neurofibromatosis type 1 were found to be significantly less likely than the other subtypes of moyamoya syndrome to have had either a clinical stroke (0.0% neurofibromatosis type 1 vs. 100.0% sickle cell, 60.0% trisomy 21, or 83.3% moyamoya disease, *p* < 0.01) or radiographic stroke (0.0% neurofibromatosis type 1 vs. 100.0% sickle cell, 60.0% trisomy 21, or 83.3% moyamoya disease, *p* < 0.01) at time of presentation. Patients with moyamoya syndrome associated with sickle cell disease were more likely to present with clinical and radiographic strokes. Additionally, patients with bilateral disease demonstrated no difference in final functional outcome compared to patients with unilateral disease (mRS 0.73 (SD = 1.33) vs. 1.29 (SD = 1.60), *p* = 0.63).

**Conclusion:**

Indirect surgical revascularization decreases stroke risk for pediatric patients with different forms of moyamoya disease and moyamoya syndrome. Additionally, these data suggest that sickle cell anemia-associated moyamoya syndrome may represent a more aggressive variant, while neurofibromatosis type 1 may represent a more benign variant.

## Introduction

Pediatric arterial ischemic stroke has an annual incidence of 1.72:100,000 [1]. Though rare, these infarcts can have profound impacts on both the patients and the families responsible for caring for them. Previous research has found that approximately 3.6–8.5% of all pediatric arterial ischemic strokes are related to moyamoya disease (MMD) or moyamoya syndrome (MMS) [1, 2].

MMD is defined as a pathology characterized by idiopathic intracranial carotid artery stenosis with associated collateral formation. MMS is a radiographically identical pathology to MMD but occurs secondary to an associated underlying condition. These two conditions have classically been treated surgically through either direct or indirect revascularization. Although prior work has segregated this pathology into unilateral and bilateral diseases by distinct demographic risk factors and radiological and biochemical findings [3–5], there has not been much work separating MMD and MMS by an underlying disease given the rarity of the condition as a whole. This can introduce the potential for confounding of results and lead to difficulties identifying clinically meaningful subpopulations and developing individualized treatment paradigms for these populations. To that end, we present our case series and analysis separating results by the underlying type of moyamoya to the growing body of literature attempting to understand and treat these disease processes.

## Methods

### Data collection

This was an Institutional Review Board-approved retrospective review of all moyamoya surgeries performed by the senior author (SNM) between 2009 and 2019. The electronic medical records were searched for the following data: demographic data, prior treatment history, presenting symptoms, radiographic data, neurological and functional status pre- and post-operatively, surgical details, complications, and follow-up data. Ischemic events were considered transient ischemic attacks (TIAs) if the symptoms lasted less than 24 h, and no sequelae were observed on MRI. Otherwise, all events were considered ischemic strokes. Clinical follow-up data, including modified Rankin Scale score (mRS) and incidence of clinical strokes, were obtained from the last available neurosurgical, neurological, or hematological note in a patient’s record. Post-operative strokes were identified through both review of these notes and interpretation of all post-operative MRIs each patient had within the hospital system. Angiographic evaluation of revascularization was determined by the Matsushima grade (defined as the percent of the ischemic area revascularized where *A* =  > 66%, *B* = 33–66%, and *C* < 33%) on each patient’s most recent angiogram.

Data were segregated for analysis in the following ways. Ischemic events were divided into those occurring peri-operatively (within 30 days of surgery) or post-operatively (after 30 days). Pre- and post-operative stroke rates were calculated by dividing the number of strokes a patient had in the time from presentation to our clinic or emergency department to surgery and the time from surgery until last follow-up, respectively. Each rate was then standardized over 365 days. Post-operative ischemic events were defined as ischemic events occurring in a surgically revascularized hemisphere. Additionally, the decision to preclude ischemic events prior to presentation to our team was made to bias the results towards the null rather than including events from patient-reported histories.

### Statistical analysis

All demographical and outcome data were analyzed using the Statistical Package for Social Sciences (IBM Corporation 2020). Categorical data were analyzed with a chi-square test, while continuous variables were analyzed using a Kruskal–Wallis test. Comparison of pre- and post-operative stroke rates was conducted with a paired-sample sign test. Statistical significance was defined as *p* < 0.05. For the purposes of this study, perioperative refers to the follow-up period within 30 days of surgery, and postoperative refers to the follow-up period after 30 days from surgery.

### Revascularization procedure

The majority of patients were revascularized with superficial temporal artery (STA)-pial synangiosis as follows. Patients were positioned supine with their head rotated so the operative site was facing up. The STA course was mapped with a Doppler, and the vessel was subsequently dissected with a combination of blunt and sharp dissection under the operative microscope. Branches from the parent vessel were coagulated and cut as needed to free the artery along with a small cuff of galea. Attention was then turned back to the scalp, and the temporalis muscle was incised. Self-retaining retractors were placed, and a craniotomy was performed. The dura was opened in a stellate fashion, and the arachnoid was opened in multiple places. Using 10–0 nylon suture, the galeal cuff of the STA was sewn to the pia in multiple locations to fix it to the surface of the brain. The dural leaflets were laid back onto the surface of the brain, and the craniotomy burr holes were widened to allow for free passage of the STA. Finally, the bone flap was plated to the skull over the STA, the temporalis muscle was reapproximated, and the scalp was closed in layers. Generally, in patients with bilateral disease, the two sides were completed in a staged fashion separated by several weeks.

For all patients with moyamoya, extra precautions are taken to mitigate stroke risk. All patients are admitted the day before surgery for IV hydration with 1.5×maintenance IV fluids, and all patients remain on aspirin up until the day of surgery. During surgery, EEG monitoring is performed, and care is taken to maintain good blood pressure and avoid any episodes of hypotension. After surgery, patients are monitored in the pediatric ICU, and patients remain on IV hydration for several days after surgery to maintain good blood pressure and avoid hypotension.

## Results

### Demographical analysis

Review of the electronic medical record identified 22 patients (40.9% male) with a mean age of 7.47 years (SD = 5.65). Of these, 16 patients carried a diagnosis known to predispose to MMS: six sickle cell anemia (SCA), five trisomy-21, and five neurofibromatosis type 1 (NF1). At the time of presentation, the mean modified Rankin Scale (mRS) was 1.05 (SD = 1.59). The majority of patients had clinical (63.6%) or radiographic (68.2%) strokes. Of note, both clinical and radiographic strokes were less frequently seen in NF1 patients (*p* < 0.01) on presentation. Children with trisomy 21 tended to present at an older age compared to other underlying causes. Additionally, 27.3% of patients were already on an antiplatelet agent at presentation to the neurosurgeon, with the remaining patients started on aspirin at the time of presentation (see Table 1).

**Table 1:** Demographical data for total cohort and underlying disease subgroups. Data are presented as counts with percentages (%) or means with standard deviation

**Patient population**	**Total cohort**	**SCA**	**Trisomy 21**	**NF1**	**MMD**	***p*** **value**
* N* (patients)	22	6	5	5	6	
* N* (hemispheres)	30	11	5	5	9	
Gender (% male)	9 (40.9%)	5 (83.3%)	1 (20.0%)	1 (20.0%)	2 (33.3%%)	0.09
Age at presentation	7.5 (5.7)	5.3 (2.8)	12.7 (7.3)	5.6 (5.3)	6.9 (5.0)	0.38
Pre-op mRS	1.05 (1.59)	1.67 (1.63)	0.60 (1.34)	0.00 (0.00)	1.67 (2.07)	0.15
Presenting symptoms						
Stroke	14 (63.6%)	6 (100.0%)	3 (60.0%)	0 (0%)	5 (83.3%)	< 0.01*
TIA	4 (18.2%)	1 (16.7%)	0 (0%)	1 (20.0%)	2 (33.3%)	0.56
Seizure	4 (18.2%)	0 (0%)	3 (60.0%)	0 (0%)	1 (16.7%)	0.04
Radiographic findings						
Radiographic strokes	15 (68.2%)	6 (100.0%)	4 (80.0%)	0 (0.0%)	5 (83.3%)	< 0.01*
Ivy sign	14 (63.6%)	4 (66.7%)	3 (60.0%)	4 (80.0%)	3 (50.0%)	0.77
Suzuki score	3.83 (1.07)	3.50 (1.43)	4.20 (0.84)	4.00 (0.71)	3.89 (0.93)	0.64

*SCA* sickle cell anemia, *NF1* neurofibromatosis 1, *MMD* moyamoya disease, *mRS* modified Rankin Scale

*p < 0.05

Thirty revascularization procedures were performed in the 22 patients. All but one patient (96.7%) underwent an indirect revascularization through either a STA-to-pial synangiosis (*N* = 28) or a frontal pericranial-to-pial synangiosis (*N* = 1); one patient underwent a direct STA-to-MCA bypass. Patients requiring bilateral revascularization were more likely to have SCA than trisomy 21 or NF1 (mean number of sides 1.8 (0.8) vs. 1.0 (0.0) and 1.0 (0.0), respectively, *p* = 0.04). Intraoperatively, there were no ischemic complications and only 13.6% of patients had changes on electroencephalography, all of which normalized with an increase in blood pressure prior to completion of surgery. There were no intraoperative differences based on underlying etiology of MMD or MMS (see Table 2).

**Table 2:** Intraoperative data and peri-operative ischemic events by hemisphere for the total cohort and each underlying disease subgroups. In this study, the peri-operative period refers to the first 30 days after surgery. Data are presented as counts with percentages (%) or means with standard deviation

	**Total cohort**	**SCA**	**Trisomy 21**	**NF1**	**MMD**	***p* value**
Intraoperative data						
Indirect bypass	29 (96.7%)	11 (100.0%)	4 (80.0%)	5 (100.0%)	9 (100.0%)	
EBL (mL)	117.5 (76.1)	121.8 (73.1)	102.5 (70.9)	106.0 (111.5)	125.3 (72.2)	0.74
EEG changes	3 (10.3%)	3 (27.3%)	0 (0.0%)	0 (0.0%)	0 (0.0%)	0.14
Intraop complications	0 (0.0%)	0 (0.0%)	0 (0.0%)	0 (0.0%)	0 (0.0%)	n.s
Peri-operative ischemic events						
Stroke	1 (3.3%)	0 (0.0%)	0 (0.0%)	0 (0.0%)	1 (11.1%)	0.49
TIA	6 (20.0%)	2 (18.2%)	0 (0.0%)	0 (0.0%)	4 (44.4%)	0.12
Death	0	0	0	0	0	

*SCA* sickle cell anemia, *NF1* neurofibromatosis 1, *MMD* moyamoya disease, *mRS* modified Rankin Scale, *EBL* estimated blood loss, *EEG* electroencephalogram

### Perioperative findings

Among the 30 operations, seven (23.3%) ischemic events occurred in the peri-operative period within 30 days of surgery. Six (20.0%) of these were transient ischemic attacks (TIAs) while one (3.3%) resulted in a permanent neurological deficit (see Table 2). Additionally, non-perfusion-related complications occurred in five operations: one wound infection, one case of wound breakdown, one seizure, one cerebrospinal fluid leak, and one acute hydrocephalus in a patient with a history of an endoscopic third ventriculostomy (ETV).

### Unilateral vs. bilateral disease

Additional stratification of patients was performed by unilateral or bilateral disease and by surgical intervention (index or second procedure). There were 15 patients with unilateral disease and seven patients with bilateral disease. No patient progressed from unilateral to bilateral disease during the study period (Table 3). Demographic analysis demonstrated that the only significant difference between patients with unilateral or bilateral disease was in the rate of stroke as a presenting symptom (46.7% for unilateral disease and 100% for bilateral disease, *p* = 0.02; see Table 5). There was no difference in the incidence of peri- or post-operative ischemic events based on unilateral or bilateral disease. In the seven patients with bilateral disease requiring contralateral revascularization, there was an increased rate of peri-operative TIAs (9.1% for index surgery and 50.0% for second surgery, *p* = 0.03) despite there being no difference in intra-operative surgical variables or rate of peri-operative strokes (see Table 4). All four TIAs were ipsilateral to the operative side and resolved without permanent symptoms.

**Table 3:** Demographical and post-operative follow-up when patients are separated by unilateral or bilateral disease. All follow-up data are presented in years. Data are presented as counts with percentages (%) or means with standard deviation

	**Unilateral disease**	**Bilateral disease**	***p* value**
Demographics			
* N* (patients)	15 (68.2%)	7 (31.8%)	
* N* (hemispheres)	15 (50%)	15 (50%)	
Gender (% male)	4 (26.7%)	5 (71.4%)	0.07
Age	7.5 (6.2)	7.4 (4.7)	0.55
Pre-op mRS	0.87 (1.51)	1.43 (1.81)	0.49
SCA	2 (13.3%)	4 (57.1%)	0.05
Trisomy 21	5 (33.3%)	0 (0.0%)	0.14
NF1	5 (33.3%)	0 (0.0%)	0.14
MMD (idiopathic)	3 (20%)	3 (42.9%)	0.33
Presenting stroke	7 (46.7%)	7 (100.0%)	0.02*
Radiographic stroke	8 (53.3%)	7 (100.0%)	0.05
Ivy sign	10 (66.7%)	4 (57.1%)	1.00
Suzuki score	3.93 (0.88)	3.71 (1.27)	0.64
Peri-operative ischemic events			
Stroke	0 (0.0%)	1 (6.7%)	1.00
TIA	1 (6.7%)	5 (33.3%)	0.17
Post-operative ischemic events			
Stroke^a^	3 (13.3%)	3 (42.9%)	0.27
TIA	0 (0.0%)	0 (0.0%)	n.s.
Outcomes			
Clinical F/up length	3.0 (2.3)	4.7 (3.5)	0.30
Radiographic F/up length	3.4 (2.2)	4.9 (3.3)	0.37
mRS	0.73 (1.33)	1.29 (1.60)	0.63
Matsushima A	81.80%	100.0%	0.16

*SCA* sickle cell anemia, *NF1* neurofibromatosis 1, *MMD* moyamoya disease, *mRS* modified Rankin Scale

*p < 0.05

^a^Strokes occurring within a previously revascularized hemisphere

**Table 4:** Intraoperative details and peri-operative (within 30 days of surgery) ischemic events by index or second surgery. Data are presented as counts with percentages (%) or means with standard deviation

	**Index surgery**	**Surgery for second side**	***p* value**
Number of hemispheres	22	8	
Intraoperative data			
EBL (mL)	116.9 (73.6)	119.1 (87.8)	0.96
EEG changes	1 (4.8%)	2 (25.0%)	0.18
Intraop complications	0 (0.0%)	0 (0.0%)	n.s
Peri-operative ischemic events			
Stroke	1 (4.5%)	0 (0.0%)	1.00
TIA	2 (9.1%)	4 (50.0%)	0.03*

*EBL* estimated blood loss, *EEG* electroencephalogram

*p < 0.05

### Surgical outcomes

**Fig. 1:**
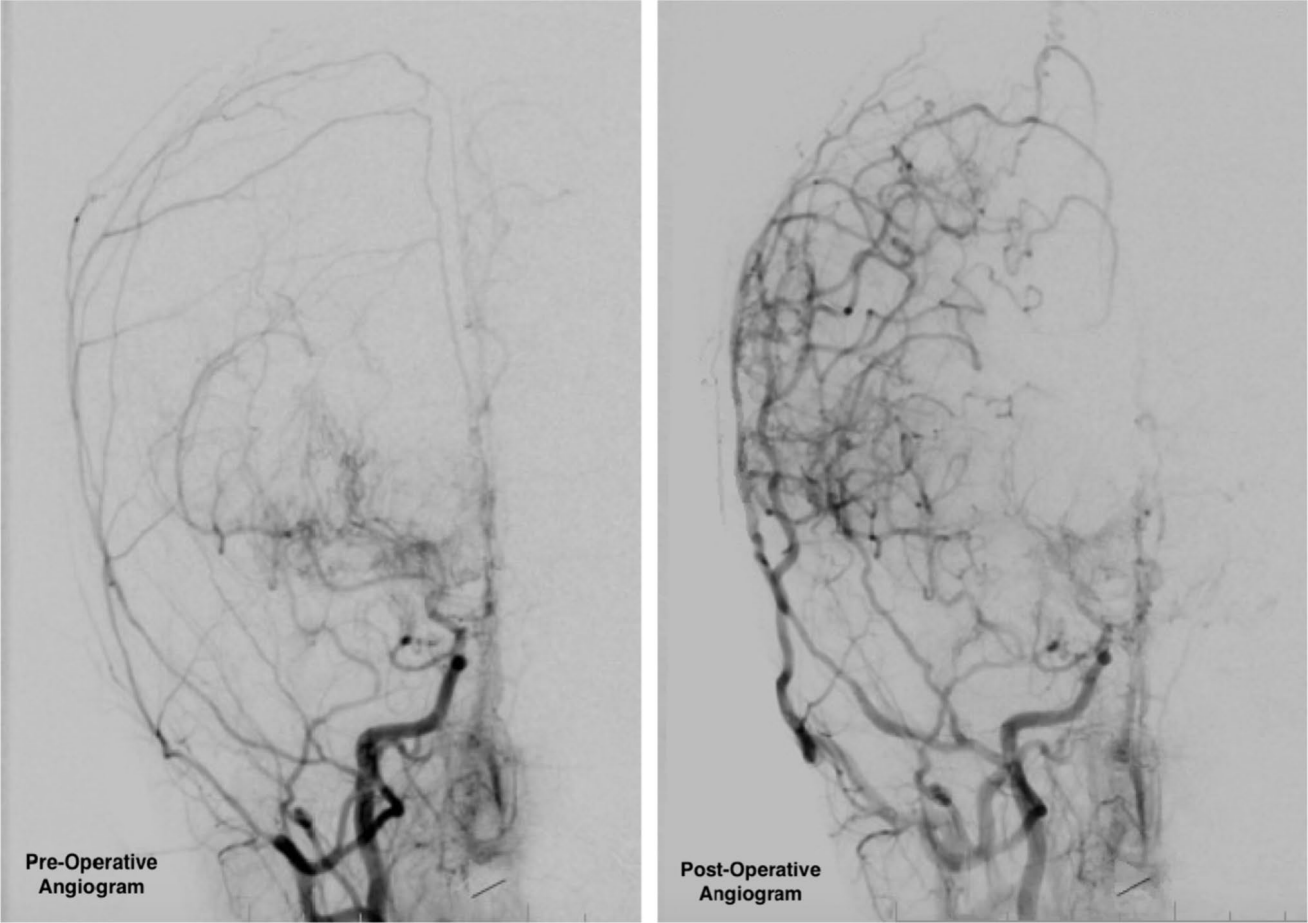
Pre- and post-operative angiograms (right common carotid artery injection) demonstrating the ischemic territory with subsequent increased vascularity following the pial synangiosis surgery

Patients were followed for a mean of 3.5 years clinically (SD = 2.8 years) and 3.9 years radiographically (SD = 2.6). During this time, there were five post-operative strokes (22.3%) on a revascularized side, two post-operative strokes (9.1%) on a non-revascularized side, and one mortality (4.5%) with no differences between underlying disease groups. There was also no difference in functional status based on etiology and the entire cohort’s functional status remained excellent throughout the follow-up period (pre-operative mRS = 1.05, SD 1.59 and post-operative mRS = 0.91, SD = 1.41, respectively, *p* = 0.38). Angiographic evaluation demonstrated successful revascularization of the ischemic territory with 90.5% of all patients achieving Matsushima A revascularization (see Table 5). A representative angiogram is demonstrated in Fig. 1.

**Table 5:** Clinical and radiographic outcome by patient for the total cohort and each underlying disease subgroup. Data are presented as counts with percentages (%) or means with standard deviation

	**Total cohort**	**SCA**	**Trisomy 21**	**NF1**	**MMD**	***p* value**
Clinical follow up						
Length	3.5 (2.8)	5.2 (3.1)	3.5 (3.0)	2.9 (2.6)	2.3 (2.2)	0.27
mRS at follow-up	0.91 (1.41)	1.33 (1.37)	0.60 (0.89)	0.00 (0.00)	1.50 (2.07)	0.18
Mortality	1 (4.5%)	0 (0.0%)	0 (0.0%)	0 (0.0%)	1 (16.7%)	0.43
Radiographic follow-up						
MRI F/up length	3.9 (2.6)	5.1 (2.9)	4.0 (3.1)	3.7 (2.2)	2.6 (2.3)	0.44
Angio F/up length	1.5 (0.7)	2.0 (0.9)	1.3 (0.2)	1.0 (0.0)	1.1 (0.2)	0.03*
Matsushima A	19 (90.5%)	6 (75%)	4 (100.0%)	3 (100.0%)	6 (100.0%)	0.31
Post-operative ischemic events						
Stroke^a^	5 (22.3%)	2 (33.3%)	1 (20.0%)	0 (0.0%)	2 (33.3%)	0.52
TIA	0 (0.0%)	0 (0.0%)	0 (0.0%)	0 (0.0%)	0 (0.0%)	n.s

*SCA* sickle cell anemia, *NF1* neurofibromatosis 1, *MMD* moyamoya disease, *mRS* modified Rankin Scale, *F/up* follow-up

*p < 0.05

^a^Strokes occurring within a previously revascularized hemisphere

Evaluation of the change in stroke rate was conducted for the total cohort and each underlying cause. The stroke rate decreased in the total cohort (2.45 strokes/year (SD = 3.63) pre-operatively and 0.23 strokes/year (SD = 0.77) post-operatively, *p* < 0.001) and for patients in each cohort (see Table 6). There was one patient who died in long-term follow-up due to severe progressive moyamoya disease.

**Table 6:** Pre- and post-operative stroke rates standardized per 365 days. Data presented as means with standard deviation

	**Pre-operative stroke rate**	**Post-operative stroke rate**	***p* value**
Total	2.45 (3.63)	0.23 (0.77)	< 0.001*
SCA	3.19 (3.10)	0.13 (0.25)	0.03*
Trisomy 21	0.73 (0.91)	0.09 (0.19)	0.25
NF1	0.00 (0.00)	0.00 (0.00)	n.s
MMD	5.19 (5.16)	0.63 (1.46)	0.06

*SCA* sickle cell anemia, *NF1* neurofibromatosis 1, *MMD* moyamoya disease

*p < 0.05

## Discussion

MMD/MMS remains a rare entity with an annual incidence of 0.09:100,000 in the United States [6]. When these diseases are separated by the underlying etiologies, however, the incidence of MMS becomes even smaller. This finding is reflected in the published literature with multiple large studies on MMD but significantly fewer and smaller reports on subcategories of MMS [7–11]. Here, we have presented our series of MMD/MMS patients segregated by underlying disease process. The results from this cohort demonstrate that indirect surgical revascularization is a safe and effective treatment for patients with MMD or MMS from SCA, trisomy 21, or NF1. While this finding adds to the growing body of literature supporting indirect revascularization for these patients [8, 11–13], several other findings warrant further discussion.

### SCA and MMS

Of all the associated conditions assessed here, patients with SCA-induced MMS appeared to have the most aggressive type of MMS. Some prior studies have shown that patients with SCA-associated MMS have high preoperative stroke risk. Newman et al. documented a stroke risk of 1 per 3.43 patient years in patients with SCA-associated MMS before surgical revascularization [14]. The prevalence of moyamoya syndrome in SCA patients has been estimated at 20–35% by cerebral angiography [15, 16]. In our study, patients with SCA-associated MMS were significantly more likely to present with prior ischemic strokes (radiographic or clinical). Additionally, they were more likely than patients in the other groups to present with bilateral involvement. Despite these issues, they responded well to indirect revascularization with a significant post-operative reduction in stroke rate.

### NF1 and MMS

Pediatric MMD/MMS classically presents the following ischemic events with published rates of stroke ranging from 67.8 to 92% [7, 11, 17]. The NF1 cohort presented here, however, was significantly less likely to have suffered a clinical or radiographic stroke prior to presentation. This is similar to more recently published reports of low pre-operative stroke rates in this population with clinical stroke rates between 0 and 43.7% and radiographic stroke rates between 6.7 and 56.3% [9, 10, 18]. Additionally, the cited higher rates of infarctions are seen predominantly in NF1 patients who have undergone prior cranial radiation [10]. While it is clear that earlier and incidental detection of MMS can be accomplished by the intensive intracranial screening that NF1 patients receive, our population of NF1 patients demonstrated no difference in age or pre-operative Suzuki score. This suggests that these patients’ MMS was not necessarily detected at an earlier state but rather was detected before signs of ischemic events. When this finding, which is supported by previous literature [10], is combined with the lack of ischemic complications seen in the peri- and post-operative period in both this cohort and previous reports [9, 18], it raises the possibility that the MMS variant associated with NF1 is a more benign pathology than MMS associated with other disease processes such as SCA. Because previous work has demonstrated that NF1-associated MMS is progressive in nature [10] and that earlier surgery is associated with improved outcomes [3, 10], surgical revascularization was still performed in our cohort without evidence of stroke, although most patients had evidence of “ivy sign” changes on MRI.

### Unilateral vs. bilateral disease

The differences between patients with unilateral MMD/MMS and those with bilateral MMD/MMS remain a clinically important distinction. While it is clear that there is a group of patients who progress from unilateral MMD/MMS to bilateral disease, determining the precise demographic [3], radiologic [4], and/or biochemical [5] factors predisposing this transition remains a subject of active research. When comparing the cohorts included here, the only difference in presentation between the groups was that patients with bilateral disease were more likely to present with a stroke than those with unilateral disease. Ultimately, this difference did not impact treatment and both groups were able to be safely revascularized with good functional outcomes, a finding was in agreement with previous literature [3, 8, 11].

It is worth noting, however, that despite no difference in patient outcome, more patients with bilateral disease had perioperative TIA’s after the second surgery than the first surgery. As we elected to stage bilateral surgeries on separate days, it is unclear if performing bilateral revascularization on the same day would have led to fewer TIA’s, or if those patients would have had TIA’s regardless of the timing of the second operation. Further research into this finding could better delineate the ideal time for subsequent revascularizations.

### Limitations

It is important to note that, as with many retrospective reviews on MMD/MMS, the small population of included patients limits the power of statistical testing. This lack of power was accepted here rather than clustering patients with MMS together to avoid obscuring differences that may exist among different etiologies of MMS as well as to avoid confounding by an underlying disease.

## Conclusion

Indirect surgical revascularization can be performed safely in pediatric patients with MMD or MMS and provide significant reduction in risk of future stroke. Additionally, these data suggest that SCA-associated MMS may represent a more aggressive variant, while NF1-associated MMS may represent a more benign form. Further study through large systematic reviews and meta-analyses into these rare populations can provide a better understanding of the disease process and produce improved treatments and outcomes.

## Data Availability

The datasets generated during and/or analyzed during the current study are available from the corresponding author on reasonable request.

## Declarations

### Disclaimer

The views expressed in this article are those of the authors and do not reflect the official policy of the Department of Army/Navy/Air Force, Department of Defense, or U.S. Government.
